# Clinician Perceptions Around Management of Sleep Problems in Children With Neurodisability

**DOI:** 10.1111/cch.70244

**Published:** 2026-02-13

**Authors:** Kasey Fullwood, Kate O'Donohue, Anne Bernard, Grace Langdon, Moya Vandeleur, Karen Waters, Jasneek Chawla

**Affiliations:** ^1^ Child Health Research Centre The University of Queensland South Brisbane Australia; ^2^ Queensland Children's Hospital South Brisbane Australia; ^3^ The University of Queensland St Lucia Australia; ^4^ Department of Respiratory and Sleep Medicine The Royal Children's Hospital Melbourne Parkville Australia; ^5^ Murdoch Children's Research Institute Melbourne Victoria Australia; ^6^ Department of Respiratory and Sleep Medicine The Children's Hospital at Westmead Westmead Australia; ^7^ Child and Adolescent Health University of Sydney Camperdown Australia; ^8^ Department of Respiratory and Sleep Medicine Queensland Children's Hospital South Brisbane Australia

## Abstract

**Introduction:**

Children with neurodisability (ND) experience higher rates of sleep disorders when compared to typically developing children. Children with ND frequently receive care from multiple medical services; sleep problems are commonly mentioned by caregivers and encountered by clinicians across various disciplines. However, the perspectives of these clinicians on managing sleep in this cohort of children are not widely understood. This study aimed to explore how clinicians from diverse paediatric fields perceive their role in the assessment and management of sleep disorders in children with ND.

**Method:**

A cross‐sectional survey involving clinicians across multiple specialty disciplines (excluding sleep medicine) from three tertiary paediatric hospitals in Australia was conducted. The survey included multiple choice questions, free text responses and case studies. Data analysis included quantitative (descriptive) statistics and thematic analysis of free text responses.

**Results:**

Ninety‐five clinicians participated. Of these, 80% of clinicians (*n* = 77) identified sleep as a significant issue in this population, with sleep problems regularly raised by families within routine consults. Clinicians reported predominantly (70%) relying on informal methods to screen for sleep, with limited use of validated screening tools. Confidence in managing sleep varied across specialties and sleep disorder presentations, with medical specialists generally confident across all sleep presentations. Clinicians reported offering treatment advice to families, while simultaneously reporting limited training and confidence. A lack of training, unclear referral pathways and time constraints were described as barriers for management.

**Conclusion:**

Non‐sleep specialised clinicians describe challenges when managing sleep in children with neurodisability. Findings support the development of national clinical guidelines to provide a more consistent approach to sleep management.

## Summary

- Our findings show variability in clinicians' confidence levels in managing sleep concerns.
- Key barriers identified include limited training, unclear referral pathways and time constraints.
- Clinicians highlighted the need for national guidelines to support a more standardised approach to sleep management in this population.
- Enhanced formal training and the development of practical resources were recommended.
- Upskilling allied health professionals in sleep‐related care may help mitigate time limitations faced by medical staff and support more effective multidisciplinary management.

## 1 Introduction

Neurodisability (ND) encompasses congenital or acquired conditions affecting the nervous and/or neuromuscular systems (Morris et al. 2013). These conditions can arise in early childhood and impact motor, behaviour and intellectual function (Ogundele and Yemula 2022). A specific diagnosis may not be identified; however, some conditions that fall under this term include (but are not limited to) genetic syndromes (Down syndrome, Prader‐Willi syndrome, fragile X syndrome), developmental disorders (attention‐deficit and hyperactivity disorder, autism spectrum disorder) neurometabolic conditions (phenylketonuria, childhood dementia syndromes) and neurological conditions (cerebral palsy). In Australia, approximately 12% of children aged 0–24 are living with a disability (Australian Bureau of Statistics 2022), with one in 10 meeting the diagnostic criteria for a neurodevelopmental disorder (Arabiat et al. 2018; McGuire et al. 2019). Globally, it is estimated that around 291 million children under 20 years are diagnosed with a mild–severe disability (Olusanya et al. 2022). Current literature estimates that at least 80% of children with ND experience chronic sleep problems (McDonald and Joseph 2019; Robinson‐Shelton and Malow 2016; Blackmer and Feinstein 2016), compared to around 30% of typically developing (TD) children (Fricke‐Oerkermann et al. 2007).

The aetiology of sleep difficulties in children with ND is multifactorial with disease‐specific factors often contributing to the likelihood of sleep problems (Ogundele and Yemula 2022; Hamilton et al. 2023). These factors can include communication difficulties, pain, sleep disordered breathing, interaction of medications, differences in internal circadian rhythms and behavioural factors (Ogundele and Yemula 2022; Olusanya et al. 2022; Gilbertson et al. 2021; Chawla et al. 2021). Alongside these, environmental, psychological and caregiver‐related factors are potential contributors. The associated negative impact of poor sleep is significant on a child's functional and behavioural outcomes, manifesting as daytime fatigue, irritability and poor concentration (Ogundele and Yemula 2022; Gilbertson et al. 2021). Additionally, sleep difficulties can have a significant burden on family members and caregivers, increasing stress and anxiety, thus resulting in daily disruption to family life (Chawla et al. 2023; Cooke et al. 2024).

Children with neurodisability require the involvement of multiple specialty disciplines in their day‐to‐day care and sleep‐related concerns are frequently reported by parents during routine assessments or evaluations of other comorbid conditions (Hulst et al. 2021; Varma et al. 2020). As a result, clinicians from various disciplines encounter children with ND who require management of sleep difficulties. However, despite this, the perspectives of non‐sleep specialists on managing these problems remain understudied. This study addresses this gap in current literature with the aim of evaluating the perspectives of clinicians from diverse paediatric disciplines around their role in assessment and management of sleep difficulties in children with ND.

## 2 Method

### 2.1 Study Design

This study was a cross‐sectional survey undertaken across three tertiary children's hospitals in Australia over a 12‐month recruitment period. This study was approved by The Children's Hospital Queensland Ethics Board (HREC/23/QCHQ/97774).

### 2.2 Participants

#### 2.2.1 Inclusion Criteria

Participants included doctors across a broad range of training levels (consultants, registrars and advanced trainees) working in paediatric specialties other than sleep medicine, as well as senior nursing staff affiliated with one of the three participating hospital sites (referred to as ‘clinicians’ through this paper). Participants needed to be directly involved in the delivery of care to children up to 18 years old with neurodisability.

#### 2.2.2 Exclusion Criteria

Clinicians accredited, or in training, in sleep medicine were excluded.

### 2.3 Study Procedure

Eligible practitioners were contacted via group mailing lists, direct contact and by promotion through internal presentations using a Redcap QR code and link by the authors. Direct contact included a study advertisement posted in staff common areas. A QR code to the study information sheet and Redcap link was provided with author (K.O.) facilitating recruitment.

### 2.4 Measures

#### 2.4.1 Study‐Designed Survey

A study specific survey (Appendix S1) was developed by the three senior authors who are paediatric respiratory and sleep specialists (M.V., K.W. and J.C.) working in each of the tertiary settings. Case scenarios were developed, using the three respiratory and sleep specialists, a junior doctor working within paediatric medicine (K.O.) and a PhD student (K.F.). Informal feedback was requested from the initial five participants and confirmed feasibility and acceptability. Data obtained from the survey included:

- Demographic information: paediatric discipline, levels of training and years of experience.
- Multiple choice and free text questions: frequency of encountering sleep problems, use of validated sleep screening tools and referral patterns.
- Three case scenarios: clinicians' general approach and confidence in assessment and management of (a) behavioural sleep disorder, (b) respiratory sleep disordered breathing and (c) complex mixed sleep disorder.

### 2.5 Statistical Analysis

Descriptive statistics were presented as frequencies and percentages for categorical variables to summarise questionnaire responses. Ineligible clinicians were identified and removed prior to analysis. For analysis, specialities were grouped into four broad discipline categories (Table 1). These categories were agreed by respiratory and sleep clinicians (M.V., K.W. and J.C.). Any missing data was identified and removed from analysis; this included incomplete questionnaires. Analysis was conducted using the R studio statistical software (R Core Team 2024).

**TABLE 1:** Grouping of specialities and number of clinicians within each speciality group.

Speciality groups (*N* = 95)	Included specialities (*N* = 95)
Medical specialties (*n* = 58, 60%)	General paediatrics (*n* = 26) Child neurology (*n* = 8) Endocrinology (*n* = 6) Paediatric rehabilitation (*n* = 4) Child development (*n* = 3) Neurodevelopmental and disability (*n* = 2) Adolescent medicine (*n* = 2) Genetics (*n* = 1) Mental health (*n* = 1) Metabolic medicine (*n* = 1) Oncology (*n* = 1) Palliative care (*n* = 1) Paediatric obesity (*n* = 1) Respiratory (*n* = 1)
Critical Care (*n* = 10, 10.52%)	Emergency department (*n* = 8) Intensive care unit (*n* = 2)
Surgical (*n* = 7, 7.36%)	Ear, nose and throat (*n* = 6) Paediatric surgery (*n* = 1)
Missing (*n* = 20)	

### 2.6 Qualitative Data Analysis

Free text survey responses from 67 clinicians were analysed. Responses were imported into Nvivo, a qualitative coding program. Thematic analysis (TA) was used to analyse the qualitative data and to report repeated patterns (Kiger and Varpio 2020). TA has six steps: (1) familiarisation of the data, (2) coding the data, (3) generating themes, (4) review and development of themes, (5) defining and refining themes and (6) writing the report (Kiger and Varpio 2020). These six steps were led by author G.L. who has training in qualitative analysis methods and an undergraduate background in psychology. Free text answers were in response to beliefs that clinicians held regarding management of sleep problems in children with ND. Specific questions included within TA analysis can be found in Appendix S2.

## 3 Results

### 3.1 Quantitative Analyses

A total of 95 clinicians were included. Demographic data are summarised in Table 2. The largest proportion of clinicians worked within metropolitan tertiary hospitals (*n* = 76, 79.2%). A range of training levels were captured with consultants comprising 59.4% of the clinicians with majority trained within Australia (76%). Individuals from a diverse range of disciplines participated with medical specialties being more commonly represented (*n* = 57, 60%). Within this group, general paediatric specialists were most common (*n* = 26, 45.6%). Demographic data suggest many participants worked within one of the three tertiary settings, alongside additional healthcare settings.

**TABLE 2:** Demographic data.

	*N*	%
Healthcare setting		
Metropolitan tertiary hospital	75	78.95
Regional secondary hospital	3	3.1
Rural hospital	2	2.1
Other (private *n* = 1, paediatric hospice *n* = 1)	2	2.1
Missing	13	13.68
Medical level		
Registrar	4	4.2
Senior trainees	6	6.2
Consultant	57	59.4
Specialist nurse (CN, CNC, nurse practitioner)	10	10.4
Missing	18	18.95
Training location		
Australia	72	75.79
United Kingdom	2	2.1
New Zealand	1	1.0
Ireland	1	1.0
Other (United Kingdom and Singapore)	1	1.0
Missing	18	18.95

Eighty percent of clinicians (*n* = 77) believed that sleep was a significant issue for children with ND and 78% (*n* = 74) of clinicians reported that they encountered sleep problems when delivering care. Sleep was often raised by clinicians within routine consults; however, 76% (*n* = 72) reported families initiated discussions on sleep within their consults. Medical specialties reported the highest likelihood to have sleep problems mentioned (*n* = 51, 96%); however, sleep was commonly mentioned to critical care (*n* = 6, 75%) and surgical (*n* = 6, 86%) clinicians. Furthermore, over half of clinicians (*n* = 53, 55%) reported their training had inadequately prepared them to manage sleep problems in this group of children.

### 3.2 Sleep Screening Tools

Fifty‐two percent of clinicians (*n* = 50) routinely screened for sleep problems. Of these, 71% did not use a standardised screening tool, instead opting to use their own version. In most cases, this was reported to be questions on sleep as part of the medical history, combined with adaptations of selected questions from established sleep screening tools. Appendix S3 summarises the screening tools that clinicians were asked to select from and any others that were mentioned in free text responses.

### 3.3 Approach to Management of Sleep Difficulties Encountered

**FIGURE 1:**
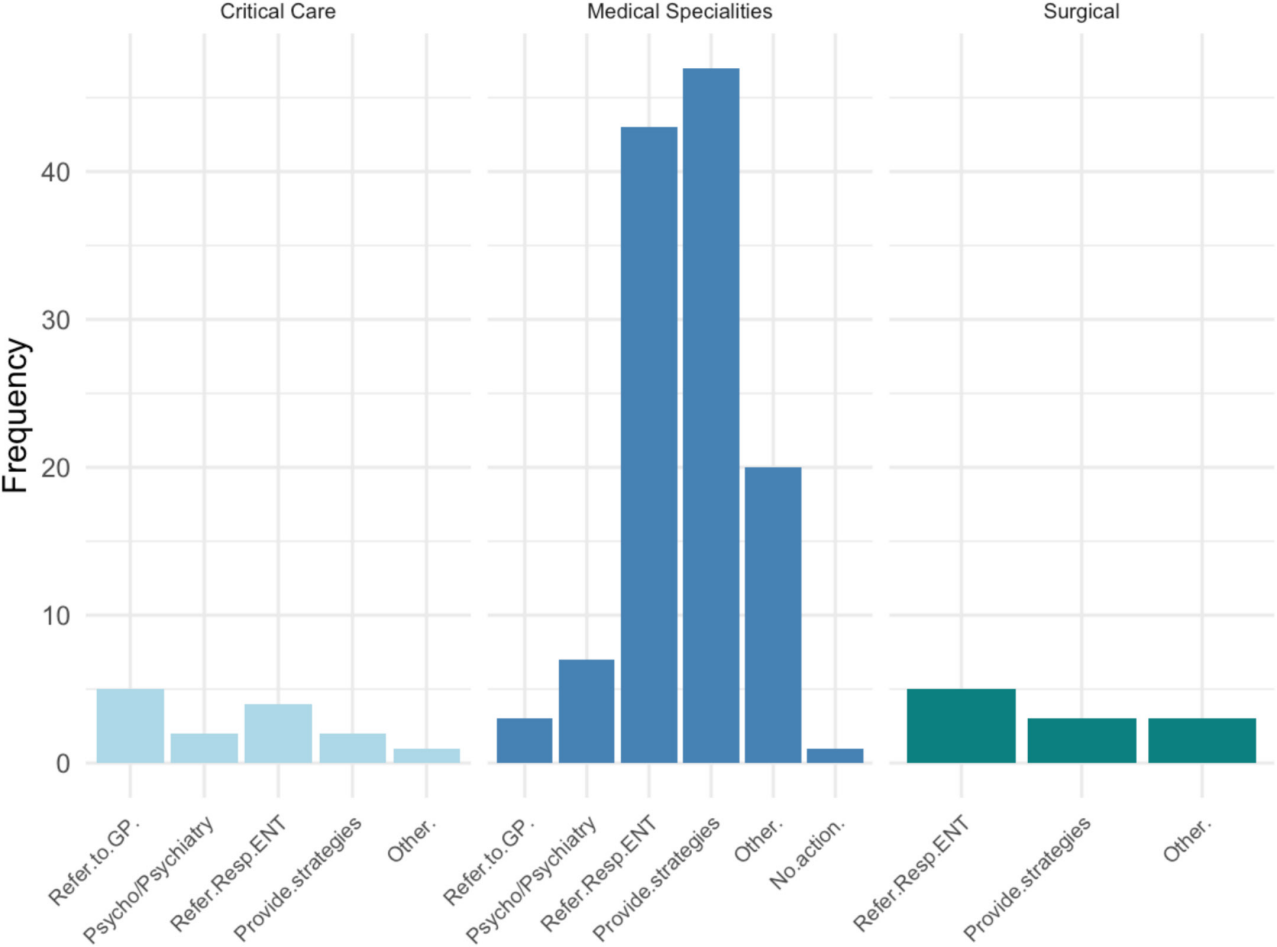
Treatment approaches for sleep problems in children with ND.

Clinicians described a combination of management strategies opposed to recommending a single treatment option. This often included a referral to another specialist (e.g., ENT, Sleep) as well as providing direct advice to caregivers themselves. Figure 1 summarises treatment approaches reported by the clinicians.

Clinicians from medical specialities were the most likely to provide strategies to families themselves (*n* = 47, 89%). Strategies consisted of recommending medication reviews, prescribing medication to improve sleep, providing sleep education resources and advising on sleep hygiene strategies. However, the degree of information provided to parents in this area varied. In contrast, clinicians from surgical specialities were more likely to refer on to sleep or ENT colleagues (*n* = 5, 71%) (inclusive of those within their own speciality). Additionally, some (*n* = 3, 43%) reported that they would suggest allergy tests, provide advice around sleep positioning or recommend specific surgical treatments and medications such as nasal sprays.

### 3.4 Case Scenarios

Three case scenarios were included to represent different sleep disorders experienced by children with ND, with clinicians grading their confidence in management. Clinicians from surgical specialties were most confident managing the scenario around a respiratory sleep disorder [Case 2] (*n* = 5, 71% confident); however, they were less confident in managing a behavioural sleep disorder [Case 1] (*n* = 6, 86% not confident). Clinicians from a critical care background were generally not confident in managing all three scenarios (Case 1: *n* = 2, 25% confident, Case 2: *n* = 4, 50% confident, Case 3: *n* = 1, 13% confident). Medical specialists reflected moderated confidence in managing all three case scenarios (Case 1: *n* = 33, 64% confident, Case 2: *n* = 29, 56% confident, Case 3: *n* = 23, 44% confident). No difference was found in the responses to which screening tools would be used or the reported treatment approaches that would be employed between clinician groups or cases. Consistent with earlier findings, clinicians were unlikely to use a screening tool for any of the case scenarios described, and management strategies commonly comprised referring onto a general practitioner or specialist and/or directly providing advice to improve sleep to families.

### 3.5 Qualitative Analyses

**FIGURE 2:**
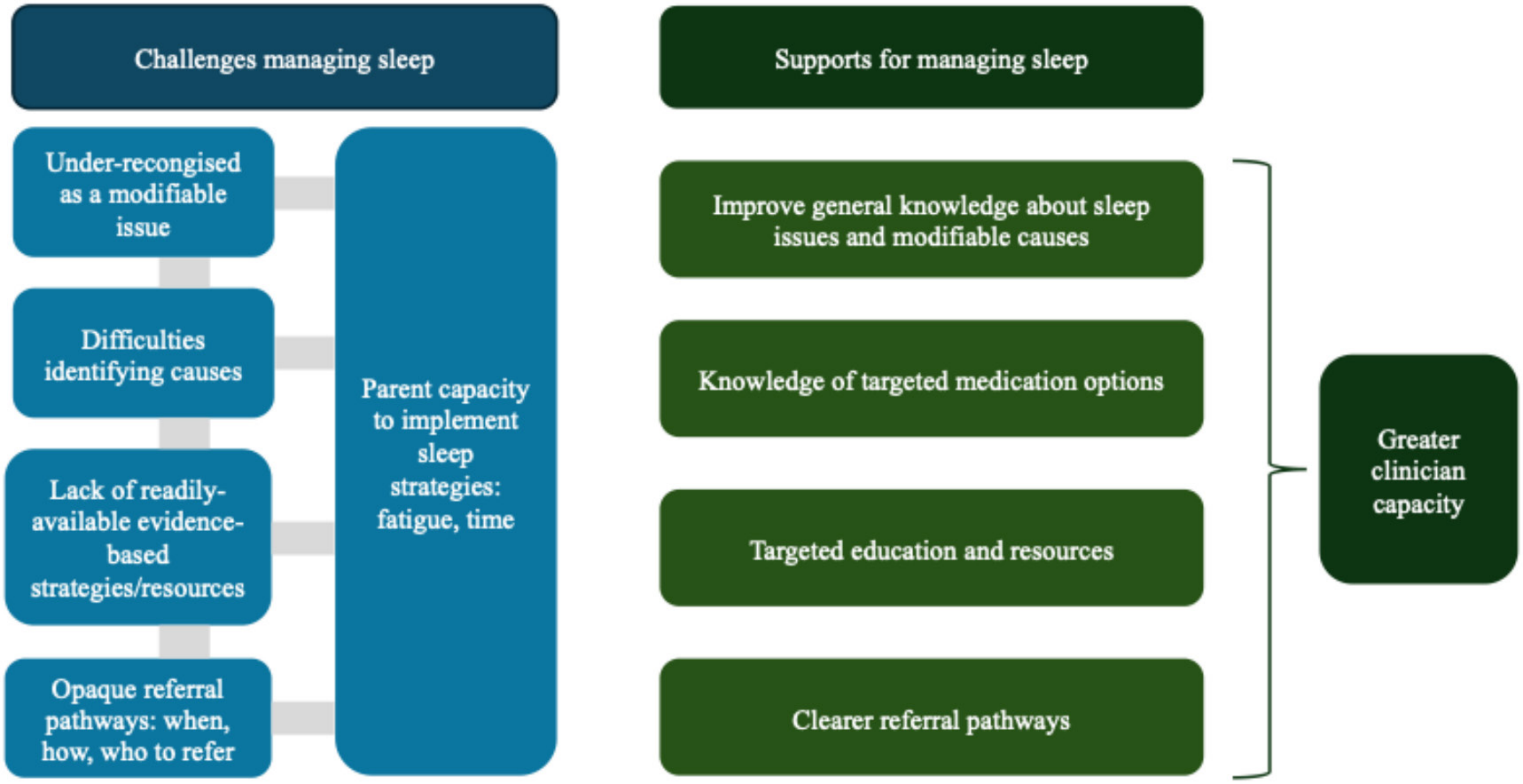
Overview of qualitative findings.

Qualitative findings are based upon the free text responses from 67 clinicians in response to questions from both parts 1 and 2. Figure 2 represents an overview of qualitative findings.

### 3.6 Perceived Challenges in Management

#### 3.6.1 Diagnosis and Referrals

Clinicians reported facing many challenges when managing sleep problems in this population. Clinicians stated sleep disorders were an ‘under‐recognised problem’ (*Neurology Consultant*) and due to the multifactorial nature of sleep problems in this population, they often found it challenging ‘identifying the cause of the issue’ (*General Paediatrics Specialist Nurse*), and thus ‘deciding when to refer to a sleep or respiratory specialist’ (*General Paediatrics Consultant*). Clinicians reported a lack of ‘evidence‐based advice’ (*Metabolic Advanced Trainee*); strategies, resources and community programs impacted their ability to treat sleep. Furthermore, a lack of streamlined approaches and confusion around sleep service referral pathways causes ‘delays in accessing specialist care’ (*Endocrinology Consultant*), with clinicians often ‘no[t] sure who to refer them to, usually suggest they need to discuss with their general paediatrician’ (*Endocrinology Consultant*).

#### 3.6.2 Collaboration With Caregivers and Care Teams

Clinicians perceived that parents often experience ‘parental fatigue or anxieties’ (*Child Development Consultant*), burnout and stress from trying to manage sleep problems. Clinicians described how this affected their approach to management, describing difficulty in balancing treatment goals, ‘parents carer burnout’ (*Palliative Care Consultant*) and ‘family capacity in applying strategies’ (*General Paediatrics Consultant*). Clinicians also identified difficulties when navigating collaborative care and the ‘complex care management and communication between the teams involved’ (*ENT Specialist Nurse*).

#### 3.6.3 Time Constraints

Time constraints within consultations and minimal ongoing care from certain departments (ED, PICU and Surgery) found addressing sleep would take away from the purpose of their consult, ‘I do not see it as something I can address in the ED’ (*Paediatrics ED Consultant*); ‘I certainly don't have the capacity to delve deeply into the issue or make recommendations’ (*Paediatrics ED Consultant*).

### 3.7 Beneficial for Management

Clinicians identified multiple areas for improvement that they perceived could benefit both clinicians and families of children with ND. First, they reported a desire for ‘better education, more resources’ (*General Paediatrics Consultant*), training and information on sleep and possible treatments, identifying improved accessibility to sleep‐related resources would help improve management. This included a focus on more education around the pathology of sleep [problems in children with ND] and a ‘structured, evidence‐based approach with escalation’ (*Endocrinology Consultant*) of sleep strategies. Second, some clinicians also noted the importance of improving knowledge in all aspects of sleep management to provide a more holistic approach ‘better understanding of causes of sleep problems and behavioural techniques to support families with this’ (*General Paediatrics Specialist Nurse*). Third, clinicians identified that a more ‘streamlined referral pathway’ (*Paediatrics Surgery Consultant*) to sleep specialists with clear guidelines on when to refer, the referral process and wait times would be beneficial for patients to help them receive earlier intervention. Finally, some clinicians reported that ‘Medication prescribed by Dr’ (*Neurology Specialist Nurse*), namely, ‘Melatonin, sometimes Clonidine’ (*General Paediatrics Advanced Trainee*), would be the most beneficial for managing sleep problems. However, some noted the need for more education and support on sleep medication as they found they lacked the ‘knowledge of acceptable medicines’ (*Paediatrics ED Consultant*) and consequently had challenges in ‘choosing the best medication’ (*Rehab Consultant*) for this population.

## 4 Discussion

This study included non‐sleep specialised medical staff from various departments in three tertiary paediatric hospitals in Australia. Our findings demonstrate that clinicians outside of sleep medicine who are involved in the care of children with ND frequently encounter sleep problems in routine care. Clinicians reported that sleep was a prominent issue, even during acute presentations in emergency departments, indicating families are seeking support in this area and their needs may be unmet.

The commonest way sleep problems were raised was through parent‐initiated discussion. Although validated screening tools exist for sleep, they were rarely used. Many clinicians relied on informal screening approaches they developed themselves. This organic approach likely derives from responding to specific concerns, rather than using broad‐based screening tools that could identify multiple sleep problems. Subsequently, it may also reflect both the perceived impracticality of lengthy questionnaires during short consultations and a limited applicability to children with ND. A lack of awareness about availability, administration and interpretation of available tools may also contribute to their limited use. This is consistent with previous research showing a lack of perceived benefit over clinical judgement and the impracticality of administration was a barrier to their use (Jensen‐Doss and Hawley 2010). At present, there is no one universally accepted tool specifically recommended for screening for sleep problems in children with ND. Therefore, an important future consideration for sleep specialists is the provision of evidence‐based national recommendations that can provide some guidance around the optimal tools for use with different clinical presentations in this population. This will require further research that determines clinically significant thresholds in ND populations for validated tools and to develop resources that support interpretation and referral decisions for non‐specialist clinicians.

Clinicians also expressed confusion around using sleep medications for children with ND. While many reported perceived benefits of sleep medications such as melatonin (prescription‐only in Australia), they simultaneously recognised their discomfort with prescribing it without understanding the underlying aetiology of the child's sleep difficulties. This likely reflects the limited evidence available to guide medication use for this population and highlights the importance of further research to avoid under or overprescribing of medication inappropriately for children with ND. Recently, recommendations for melatonin use in typically developing children (Force et al. 2025) and children with autism and neurogenetic conditions (Kotagal et al. 2024) were released; however, a gap remains in how these findings apply to other ND conditions.

Clinicians provided varying degrees of advice and support to caregivers, despite indicating feeling unequipped to manage sleep problems. This raises concern around the effectiveness of the advice provided for families and is consistent with recent findings on the inconsistencies of sleep management approaches (Fullwood et al. 2025). Barriers described included gaps in knowledge, time constraints within standard consultations, uncertain referral pathways and prolonged wait times to access specialist care. A self‐reported lack of formal training in paediatric sleep management was a recurring theme across clinician groups, alongside being unaware of resources and community‐based programs to refer to families for further support. This lack of formal sleep education during professional training programs is a consistent finding within the literature (Olusanya et al. 2022; Mindell et al. 2013) and highlights the need for targeted education and resource provision to improve care. While these problems were raised by Australian clinicians, these challenges are faced globally. Worldwide, there is a demand for enhanced sleep education programs in order to address the multifaceted challenges seen in paediatric sleep and, more specifically, paediatric ND sleep (Spruyt et al. 2025).

Despite challenges, clinicians also proposed potential solutions. First, they expressed a strong desire for national guidelines to provide a standardised approach to guide diagnosis, investigation and treatment in a timely manner. Such guidelines could improve the use of questionnaires that cover a broad range of sleep issues which would be relevant to the primary care providers in the community. Second, clinicians acknowledged that they not only required focused training on sleep health specific to children with ND themselves, but that other health care providers who regularly provide care to children with ND also needed to be engaged. Clinicians recognised that they experience significant time constraints in their clinical consultations, making it difficult to prioritise addressing sleep. It is possible that other providers, such as psychologists, physiotherapists and occupational therapists may have greater capacity to engage with families around sleep disruption and importantly, may also provide additional beneficial perspectives on management. This is an important consideration for future models of care, particularly as allied health professionals are often heavily involved in supporting families of children with ND and develop a strong rapport through more frequent appointments and longer consultations. This potentially provides an ideal setting within which to integrate pro‐active screening and support for sleep difficulties reported by children with ND and their families. Upskilling this key group of health professionals to evaluate sleep and deliver tailored sleep interventions for children with ND may assist in addressing sleep difficulties.

A key strength of the study is the diversity of participating clinicians, who represent a broad range of specialties across three major tertiary centres in Australia. This diversity enhances the relevance of findings to other care settings. However, limitations are acknowledged. While a range of specialities were represented, some specialities only had a small number of respondents. As a result, their views may have been underrepresented in comparison to those from more represented groups, such as general paediatricians, who made up a substantial proportion of the sample. This may have skewed the findings towards their perspective and may also mean that only those who are regularly encountering sleep difficulties in children with ND and perceive this as relevant to their practice have responded. Additionally, the study did not capture the perspectives of clinicians from rural or regional settings, where access to specialised services is often limited. Understanding the unique challenges faced by these clinicians who are remote from tertiary specialists is essential to ensure any guidelines developed are feasible and relevant to all practitioners, regardless of location. Further, the study procedure limited the ability to calculate a denominator for the survey and may represent a self‐selection bias limiting the generalisability of the results.

## 5 Conclusion

In summary, the findings of this study provide strong justification for why the development of national clinical guidelines for the management and assessment of sleep problems in children with ND needs to be a priority. Given the limits of evidence‐based medicine in some aspects of the management of sleep in this population, establishing consensus on the most effective strategies, stepwise escalation of strategies, appropriate referral pathways and identifying relevant resources may represent the initial steps of developing these guidelines. Consensus‐based methodologies such as the Delphi method offer a framework for guideline development, allowing for the systematic collection of expert opinion in fields where evidence is still emerging (Murphy et al. 1998). Engaging sleep specialists in such research is essential as their expertise will be key in guiding the formulation of recommendations to support non‐sleep specialised clinicians and will ensure the complex sleep needs of children with ND can be optimally met.

## Author Contributions

**Kasey Fullwood:** conceptualization, investigation, writing – original draft, methodology, validation, writing – review and editing, visualization, project administration. **Kate O'Donohue:** conceptualization, investigation, writing – original draft. **Anne Bernard:** data curation, formal analysis. **Grace Langdon:** data curation, formal analysis. **Moya Vandeleur:** conceptualization, methodology, writing – review and editing. **Karen Waters:** conceptualization, methodology, writing – review and editing. **Jasneek Chawla:** conceptualization, funding acquisition, methodology, supervision, writing – review and editing.

## Funding

This research was supported by the National Health and Medical Research Council, Australia, through a Medicine Research Future Fund grant (Application ID 2018007).

## Ethics Statement

This study was approved by The Children's Hospital Queensland Ethics Board (HREC/23/QCHQ/97774).

## Consent

Written consent was obtained from all involved participants.

## Conflicts of Interest

The authors declare no conflicts of interest.

## Supporting information


**Appendix S1:** Supporting information.

**Appendix S2:** Supporting information.

**Appendix S3:** Supporting information.

## Data Availability

Data are available on request to the corresponding author.
