# Supplementary material for: Clinician Perceptions Around Management of Sleep Problems in Children With Neurodisability
Source: Child Care Health Dev. 2026 Feb 13;52(2):e70244. doi: 10.1111/cch.70244 (PMC12903188; doi:10.1111/cch.70244)
Supplement: Supplementary file 1 — Appendix S1: Supporting information. [file CCH-52-e70244-s003.docx]

**A Questionnaire for Clinicians**

**Clinician Perceptions Around Management of Sleep Problems in Children with Neurodevelopmental Disorders.**

1. Which health facility are you based in?

1. Type answer

2. What speciality do you work in?

1. Type answer

3. What is your level?

1. Specialist nurse (CN, CNC, nurse practitioner)

2. Registrar

3. Advanced trainee

4. Fellow

5. Consultant

4. How long have you worked in this role?

1. <1 year

2. 1-5 years

3. 5-10years

4. >10 years

5. Where did you complete the majority of your post-graduate training?

1. Australia

2. Overseas (please specify ________)

6. Have you encountered sleep problems in children with ND when delivering care to this population?

1. Yes

2. No

7. If yes, was this topic raised by family or did you raise this as part of your routine consult?

1. Raised by family

2. Part of routine consult

8. How often do families of children with ND mention sleep problems in your consults?

1. Always

2. Sometimes

3. Rarely

4. Never

9. Do you believe sleep problems are a significant issue in children with neurodisability?

1. Yes

b. No

10. Do you believe your training has adequately prepared you to address sleep problems in children with ND?

a) Yes

b) No

11. Do you routinely screen for sleep problems in children with ND?

c) Yes

d) No

12. Which screening tool do you use?

a) None

1. Paediatric Sleep Questionnaire
2. Modified Simons & Parraga Sleep Questionnaire (MSPSQ)
3. Modified Epworth Sleepiness Scale
4. Children’s Sleep Habits Questionnaire (CSHQ)
5. BEARS sleep screening tool
6. Other (please specify)_______________________

13. If sleep is a big problem raised during your consult, what is your approach? *Tick all that apply*

a) Refer to GP

b) Arrange psychology/psychiatry support

c) Provide strategies for families – if yes please specify

d) Refer to other specialties – i.e. respiratory, ENT

e) No action

f) Other – please specify

13. What do you find the most challenging when managing sleep problems in children with ND?

1. Please specify

14. What do you believe will provide the most benefit in improving management of sleep problems in this cohort?

i. Please specify

**Case based questions:**

**Scenario 1: non-respiratory**

Within a follow-up consult, a parent mentions their child (6 years old with ADHD) is having issues failing asleep at night. The parents are spending lengthy amounts of time in the child’s bedroom assisting them in falling asleep. The child regularly awakens throughout the night and climbs into bed with the parents. The child is fatigued in the morning with it impacting their behaviour throughout the day. The parents are visibly exhausted and desperate for guidance.

1. Would you utilise screening tools to diagnosis this issue?

1. Yes

2. No

2. If yes, which tool would you use

1. Paediatric Sleep Questionnaire

2. Modified Simons & Parraga Sleep Questionnaire (MSPSQ)

3. Modified Epworth Sleepiness Scale

4. Children’s Sleep Habits Questionnaire (CSHQ)

5. Other (please specify)_______________________

3. If no, why not?

1. Insufficient time to use screening tool

2. Lack of awareness of which screening tools to use

3. Lack of knowledge on how to apply screening tool to guide further investigation/management

4. Not aware of specific screening tools used to identify sleep problems in children with neurodisability

5. Other – *please specify*

4. Would you feel confident in managing this sleep issue?

1. Yes

2. No

5. How would you manage this? *Select all that apply*

1. No management

2. Refer on – *please list all ______*

3. Medication – *please list*

4. Parent education/advice

5. Other – *please describe*

**Scenario 2:**

A parent of a child with a complex disability mentions they have noticed their child has started snoring throughout the night. They explain their child appears sleepy throughout the day despite getting a full nights rest.

1.Would you utilise screening tools to diagnosis this issue?

1. Yes

2. No:

2 If yes, which tool would you use

If yes, which tool would you use

1. Paediatric Sleep Questionnaire

2. Modified Simons & Parraga Sleep Questionnaire (MSPSQ)

3. Modified Epworth Sleepiness Scale

4. Children’s Sleep Habits Questionnaire (CSHQ)

5. BEARS sleep screening tool

6. Other (please specify)_______________________

3. If no, why not?

1. Insufficient time to use screening tool

2. Lack of awareness of which screening tools to use

3. Lack of knowledge on how to apply screening tool to guide further investigation/management

4. Not aware of specific screening tools used to identify sleep problems in children with neurodisability

5. Other – *please specify*

4. Would you feel confident in managing this sleep issue?

1. Yes

2. No

6. How would you manage this? *Select all that apply*

1. No management

2. Refer on – *please list all ______*

3. Medication – *please list*

4. Parent education/advice

5. Other – *please describe*

**Scenario 3:**

A parent mentions their child with cerebral palsy (GMFCS 4-5) frequently wakes during the night. The parents do not know why they keep waking up and have come to you for advice. How would you explore the cause of their frequent night-time awakenings?

1. Would you utilise screening tools to diagnosis this issue?

2. Yes

2. No

2. If yes, which tool would you use

3. Paediatric Sleep Questionnaire

4. Modified Simons & Parraga Sleep Questionnaire (MSPSQ)

4. Modified Epworth Sleepiness Scale

7. Children’s Sleep Habits Questionnaire (CSHQ)

8. BEARS sleep screening tool

9. Other (please specify)_______________________

3. If no, why not?

1. Insufficient time to use screening tool

2. Lack of awareness of which screening tools to use

3. Lack of knowledge on how to apply screening tool to guide further investigation/management

4. Not aware of specific screening tools used to identify sleep problems in children with neurodisability

5. Other – *please specify*

4. Would you feel confident in managing this sleep issue?

1. Yes

2. No

5.. How would you manage this? *Select all that apply*

1. No management

2. Refer on – *please list all______*

3. Medication – *please list*

4. Parent education/advice

5. Other – *please describe*

**FUTURE RESEARCH:**

Would you be interested in taking part in future research related to this topic (e.g. interview)? – *tick here*

If yes, please provide a contact email or phone number (you are not required to provide this information if you do not want to participate.
