# Supplementary material for: Clinician Perceptions Around Management of Sleep Problems in Children With Neurodisability
Source: Child Care Health Dev. 2026 Feb 13;52(2):e70244. doi: 10.1111/cch.70244 (PMC12903188; doi:10.1111/cch.70244)
Supplement: Supplementary file 2 — Appendix S2: Supporting information. [file CCH-52-e70244-s001.docx]

Questions included within qualitative analysis.

"Briefly describe the strategies you may provide to families,"

"Please specify what other approaches you may take,"

"what do you find most challenging when managing sleep problems in children with ND?" "What do you believe will provide the most benefit in improving management of sleep problems in the cohort?"

**case-based scenarios**: 1) Non-respiratory: Behavioural, 2) Respiratory: Sleep Disordered Breathing, 3) Complex disability: Cerebral Palsy (CP). Questions analysed were,

"Please specify which other tool you would use,"

"Please list medication you would prescribe,"

"Please list what parent education/advice you would provide,"

"Please specify which other reason you wouldn't use a screening tool,"

"Please list which medical or surgical specialities (i.e., sleep physician, ENT) and/or allied health specialities you would refer to."
