# Supplementary material for: Clinician Perceptions Around Management of Sleep Problems in Children With Neurodisability
Source: Child Care Health Dev. 2026 Feb 13;52(2):e70244. doi: 10.1111/cch.70244 (PMC12903188; doi:10.1111/cch.70244)
Supplement: Supplementary file 3 — Appendix S3: Supporting information. [file CCH-52-e70244-s002.docx]

List of Screening Tools and responses for ‘other’ screening tools

Paediatric Sleep Questionnaire

Modified Simons & Parraga Sleep Questionnaire (MSPSQ)

Modified Epworth Sleepiness Scale

Children’s Sleep Habits Questionnaire (CSHQ)

BEARS sleep screening tool

OSA11

Clinical history

And my own questions
My version
Questions from PSQ, CSHQ and Epworth pending on responses given by child

Modified PSQ

Sleep Diary

My version
